# Associations between 10‐year depressive symptom trajectories, cognitive function, and self‐management capacity skills among older primary care patients

**DOI:** 10.1002/alz.088300

**Published:** 2025-01-03

**Authors:** Abigail Vogeley, Patrick Cecil, Lauren Opsasnick, Laura M Curtis, Julia Yoshino‐Benavente, Morgan Bonham, Andrea Russell, Rachel O'Conor, Michael S Wolf, Rebecca Lovett

**Affiliations:** ^1^ Northwestern University, Chicago, IL USA; ^2^ Northwestern University Feinberg School of Medicine, Chicago, IL USA

## Abstract

**Background:**

Chronic depression impacts the ability to successfully manage one’s health, yet mechanisms underlying this relationship are not fully understood. Cognitive dysfunction, or impairment, may result from persistent depression. We aimed to determine whether chronic depressive symptoms, leading to poor cognition, impacted self‐management capacity among older adults with chronic conditions.

**Method:**

Data from 369 older adults from Chicago, IL and participating in a longitudinal cohort study on aging was used for this analysis. The Patient‐Reported Outcomes Measurement Information System (PROMIS) Depression 8‐item survey was administered across 4 time points over 10 years; raw scores were transformed into T‐scores. Group‐based trajectory modeling was used to identify latent depressive symptom trajectory groups. The Comprehensive Health Activities Scale (CHAS) was used to measure chronic disease self‐management skills, including comprehension and recall of print, spoken, and multimedia information. A factor score representing fluid cognitive function was derived from 11 measures across 4 cognitive domains (processing speed, working memory, inductive reasoning, long‐term memory). Demographic‐adjusted multinomial linear regressions were used to assess associations between depressive symptom trajectory groups and self‐management skills, first without and then including fluid cognitive function.

**Result:**

Mean participant age was 71 years (SD 5.3); most participants were female (71%) and non‐Hispanic white (54%). 86% had 2+ chronic medical conditions (mean 3.3 (SD 1.8)). Three distinct depressive symptom trajectories emerged: participants displayed either consistently minimal (42%), normative (46%), or persistently elevated (12%) symptoms. In multivariable analyses, participants in the persistently elevated group had poorer self‐management performance (β = ‐6.4; 95% CI: ‐11.1, ‐1.7; p = 0.007). Adjusting for cognitive function attenuated this association to a level of non‐significance, while a strong association emerged between cognition and self‐management skills (β = 16.1; 95% CI: 14.4, 17.8; p<0.001).

**Conclusion:**

Persistent depression may reduce self‐care capacity, likely due to poorer cognitive function. Improved understanding of how depression may alter cognitive function or lead to its progressive decline, and ultimately impact self‐care capacity, may inform future health system interventions to support the management of comorbid conditions.

